# Investigating the functional role of the oestrogen receptor in LY2 endocrine resistant breast cancer cells

**DOI:** 10.1186/1753-6561-9-S7-A27

**Published:** 2015-10-27

**Authors:** Chrisen Ramkaran, Alacoque Browne, Leonie Young

**Affiliations:** 1 Royal College of Surgeons in Ireland, Dublin, Ireland; 2 Endocrine Oncology Research Group, Dept of Surgery, Royal College of Surgeons in Ireland, Dublin, Ireland

## Background

The issue of acquired resistance to breast cancer regimes such as tamoxifen continues to negatively affect clinical outcomes. While many mechanisms of resistance have been discovered [1,2], there is evidence now of acquired resistance through adaptation of the oestrogen receptor itself leading to tumour progression [3]. A thorough understanding of the processes involved in the receptor's adaptation remains unclear. This study gives evidence of the gene signalling which shed light on the mechanism of adaptation in an LY2 endocrine resistant cell line oestrogen receptor knockdown model.

## Methods

Real time PCR examines the gene expression profile of the LY2 cells under various treatments including oestrogen, tamoxifen and a mixture of both.

## Results

It was found that the normal oestrogen receptor target genes PS2 and GREB1 display reduced expression without the presence of the receptor. However EGR3 signals excessively despite having the receptor stably knockdown.

## Conclusions

As a result these data provides evidence that EGR3 is involved in the adaptation of the oestrogen receptor and that global signalling of common target genes does not occur when the receptor adapts. Hence it demonstrates an initial clue of the process of adaptation in resistant tumours that have changed their receptor status.
